# A Profile of Biomass Stove Use in Sri Lanka

**DOI:** 10.3390/ijerph9041097

**Published:** 2012-03-27

**Authors:** Myles F. Elledge, Michael J. Phillips, Vanessa E. Thornburg, Kibri H. Everett, Sumal Nandasena

**Affiliations:** 1 RTI International, 3040 Cornwallis Road, Research Triangle Park, NC 27709, USA; Email: mjp@rti.org (M.J.P.); thornburg@rti.org (V.E.T.); keverett@rti.org (K.H.E.); 2 National Institute of Health Sciences, Ministry of Health, Kalutara 12000, Sri Lanka; Email: sumalnandasena@gmail.com

**Keywords:** biomass stove, indoor air pollution, Sri Lanka

## Abstract

A large body of evidence has confirmed that the indoor air pollution (IAP) from biomass fuel use is a major cause of premature deaths, and acute and chronic diseases. Over 78% of Sri Lankans use biomass fuel for cooking, the major source of IAP in developing countries. We conducted a review of the available literature and data sources to profile biomass fuel use in Sri Lanka. We also produced two maps (population density and biomass use; and cooking fuel sources by district) to illustrate the problem in a geographical context. The biomass use in Sri Lanka is limited to wood while coal, charcoal, and cow dung are not used. Government data sources indicate poor residents in rural areas are more likely to use biomass fuel. Respiratory diseases, which may have been caused by cooking emissions, are one of the leading causes of hospitalizations and death. The World Health Organization estimated that the number of deaths attributable to IAP in Sri Lanka in 2004 was 4300. Small scale studies have been conducted in-country in an attempt to associate biomass fuel use with cataracts, low birth weight, respiratory diseases and lung cancer. However, the IAP issue has not been broadly researched and is not prominent in Sri Lankan public health policies and programs to date. Our profile of Sri Lanka calls for further analytical studies and new innovative initiatives to inform public health policy, advocacy and program interventions to address the IAP problem of Sri Lanka.

## 1. Introduction

Biomass fuel is the main source for cooking for the majority of Sri Lankan households, and the heavy use of wood-burning stoves in Sri Lanka is the leading contributor to indoor air pollution (IAP) [1,2,3]. High concentrations of indoor air pollutants represent a significant health issue for Sri Lanka; comparative research in other countries has confirmed that IAP from solid fuel use is a major cause of premature deaths as well as acute and chronic diseases. This issue, however, is not broadly researched or prominent in Sri Lanka public health policies and programs. Our profile of Sri Lanka reviews what is known about biomass stove use and environmental health in-country, and calls for further analytical study to inform new public health policy, advocacy and program interventions. 

As in many countries, biomass stove use in Sri Lanka is most common in rural areas and the country’s rural estates. Over 78% of households nationwide burn wood in biomass stoves; 84% of these households are in rural areas, and 96% are households on rural estates. Even in urban areas, over 34% of the population uses wood as their main fuel source. Although more than 80% of the Sri Lankan households have electricity, it is used primarily for lighting, and wood is used for cooking [4].

Biomass stove use is most prevalent among rural and poor populations, and both of these conditions present significant barriers to the use of more efficient and safer stoves and cleaner fuels. The rural poor are the least likely to use cleaner fuels or a ventilated kitchen because the costs are prohibitive. In addition, Sri Lanka’s census data indicate the higher the education level, the lower the observed use of wood. Approximately 65% of females who have an education level higher than grade 10 use biomass fuel, while 95% of the females who have only primary schooling or an even lower level of education use biomass fuel [1].

Women and children, in particular, have a higher risk of IAP-related problems because typically they are cooking or present in the home during meal preparations, which means they are also more exposed to the airborne particles produced by burning biomass. The elderly as well are at increased risk for developing cataracts, heart disease and respiratory diseases from these airborne particles. 

Sri Lanka’s population demographic trends show both a large young population and a sizeable aging population—unusual in many developing countries—which suggests that IAP is a serious risk for large segments of the population that are indoors during cooking stove operations. Other demographic and health indicators appear in Table 1. Further, other public health problems (*i.e*., such as severe malnutrition, vaccine preventable diseases, water borne diseases, malaria, AIDS etc.) which are usually high prevalent in most of the developing countries and coincide with IAP issues are relatively low in Sri Lanka. 

**Table 1:** Sri Lanka Demographic and Development Indicators.

Population	20.5 million (2009)
Area	25,332 square miles
Race/ethnicity	82% Sinhalese, 4.3% Sri Lanka Tamil, 5.1% Indian Tamil, 7.9% Sri Lanka Moor, 0.2% Burgher, 0.3% Malay, 0.2% Other (2001)
Percent children aged 6–14 not attending school	6.3% (2001)
Adult literacy rate	92.2% (male), 89.2% (female) (2001)
Annual population growth rate	1.1% (2007–2009)
Percent population in urban areas	15% (2008)
Percent of population below national poverty line	15% (2007)
Under 5 mortality rate per 1000 live births	15 (2008)
Infant mortality rate per 1000 live births	10 (urban), 19 (rural), 29 (estates) (1996–2006)
Percent prevalence of underweight children	16.5 (urban), 21.2 (rural), 30.1 (estates) (2007)
Maternal mortality rate per 100,000 live births	39 (2008)

Sources: Asian Development Bank (ADB) Basic Statistics (2010); World Bank World Development Indicators Online (2010); Demographic Health Survey (DHS) (2007; 1996–2006); Census of Population and Housing (2001).

A higher percentage of Buddhists use biomass stoves compared to those practicing other religions, and a higher percentage of Sinhalese use biomass stoves compared to other ethnicities [5]. Further research into stove use pattern and how it differs by religion and ethnicity may lead to understanding cooking and lifestyle practices, an important step for accurately reflecting cultural factors in the development of public health advocacy and improved stove programming. Experience from other countries shows that biomass stove research and community-based initiatives that have targeted the introduction of new and improved stoves often failed to adequately account for lifestyle and cultural practices in research and program design. Despite Sri Lanka’s well-established public health system and relatively good public health indicators compared to other developing countries, limited literature suggests that the IAP issues are poorly studied to date. Synthesizing the available information on biomass stove use is an important essential first step to identify gaps, impact, and feasible interventions to address the biomass cook stoves in Sri Lanka. 

## 2. Methodology

We conducted a review of published literature, government data sources, and reports regarding Sri Lanka and biomass fuel use. Online literature searches were made via PubMed and ScienceDirect. PubMed search terms included the words “Sri Lanka” and “indoor air pollution,” “respiratory,” “cooking” and “biomass,” which yielded 117 results and eight relevant articles. The same terms were used for the ScienceDirect search and yielded more than 1800 results but only 33 relevant articles. Government data sources included Sri Lanka’s Annual Health Statistics, the Census of Population and Housing, and the Demographic Health Survey (DHS), which is a nationally representative survey used to assess the nation’s health against Millennium Development Indicators. Other sources of data included World Health Organization (WHO) statistics and reports. 

**Figure 1:**
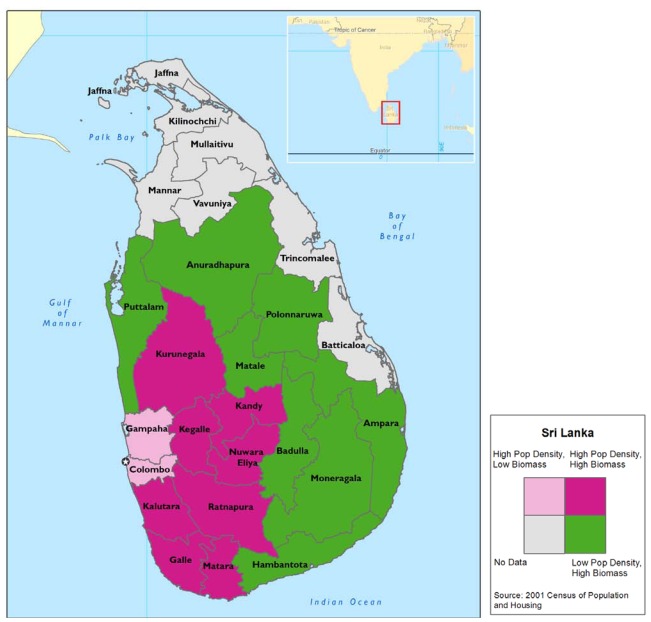
Sri Lanka population density and percentage of households using Biomass, by district-2001.

We utilized data from the 2001 Census of Population and Housing to produce a bivariate map (Figure 1) that illustrates population density and households using firewood. The 2001 Census of Population and Housing have conducted and provided the data only for 18 districts out of 25 districts of the country (the data unavailable districts are shown in gray color on the map). We used the national average values (based on Census 2001) of biomass use (80.0%) and population density (300 km^2^) to categorize (low or high) and illustrate the districts level variation in biomass use pattern. The districts were categorized into groups as “high population density and high biomass use”, “high population density and low biomass use”, and “low population density and high biomass use”. Districts of Colombo and Gampaha were identified as “high population density and low biomass use” while there were no “low population density and low biomass use” districts. 

**Figure 2:**
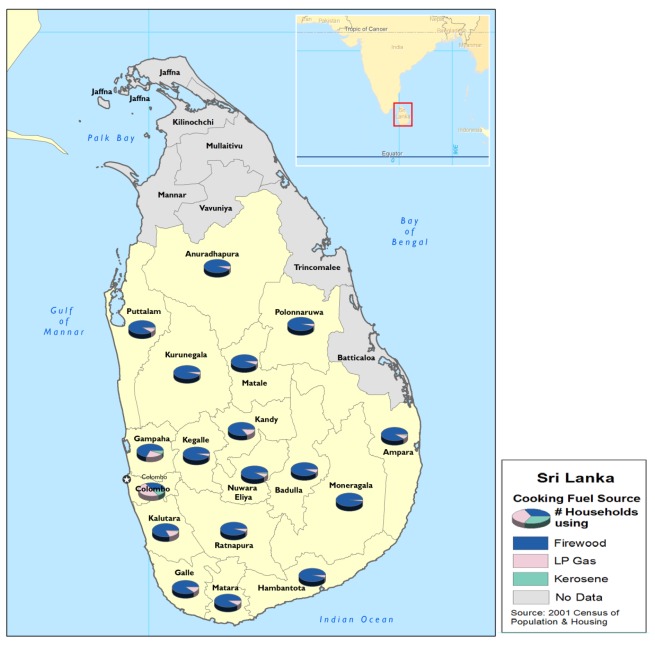
Sri Lanka cooking fuel source by district-2001.

The same source of data was used to produce Figure 2, a map of cooking fuel type by district. Pie charts on each district indicate the general proportion of households use biomass, liquid propane gas (LPG) or kerosene.

## 3. Results and Discussion

### 3.1. Profile of Biomass Fuel Use

Biomass use in Sri Lanka is limited to wood while coal, charcoal, cow dung are not used [1,6]. Crop residues may be served as alternative cooking fuel sources where wood is not readily available. Although over 80% of Sri Lankan households have electricity, this energy source is used for lighting because it is too expensive to use as cooking fuel. Even in urban sectors, where electricity, LPG and kerosene are more likely to be used for cooking, their use is largely confined to boiling water [7].

Populations in less densely populated districts are more likely to use wood as their cooking fuel source than those in the more densely populated districts. Figure 1 demonstrates that the more urban a district is, the more likely its population is to use cooking fuel sources other than wood. 

Figure 2 clearly indicates that far more Sri Lankan households use wood than any other fuel source. The figure also illustrates that those in urban and higher household income areas, such as Colombo, use more expensive fuel sources such as LPG and kerosene. 

Within Sri Lankan homes, the majority of cookstoves used are either three stones or semi-enclosed stove types. The “Anagi” is an improved cookstove made of clay that has been widely disseminated since the 1990s and is still used today [8]. A national estimate of cookstove types across the entire population is unavailable. Emissions from the improved cookstoves in Sri Lanka are not available with scientifically rigorous methodologies. 

Approximately 65% of the households using biomass fuel cook inside their main household structure, while only 9% have a separate building for cooking [5]. About 72% of the households using biomass cooking stoves have a chimney in their house/kitchen [5]. These stoves’ thermal efficiency is very poor, and there are significant quantities of emissions released into poorly vented indoor settings. Kitchens in which wood was used with traditional stoves have reported average 24 h PM_2.5_ (particulate matter with a diameter smaller than 2.5 microns) concentrations exceeding 1200 µg/m^3^ [9]. It is clear that the emissions from these types of stoves are widespread and significant IAP sources in Sri Lanka. 

Biomass stove emissions influence Sri Lanka’s IAP more than any other factor. Tobacco use is lower in Sri Lanka than other Asian countries. Among adults (≥15 years old), tobacco usage rates are 16.5% for both sexes combined. Among adolescents (13–15 years old), usage is 9.1% [10]. However, it is critical that researchers capture smoking and second-hand smoke exposure data to reduce confounding that may lead to exposure misclassification. 

### 3.2. Determinants of Biomass Fuel Stove Use

Poverty is one of the main determinants of biomass fuel use. The DHS 2007 provided the distribution of households by the wealth index. (The wealth index is a socioeconomic indicator and a proxy for the household’s long-term standard of living. It is based on data on the household’s ownership of consumer goods, dwelling characteristics, type of drinking water, toilet facilities and characteristics that are related to a household’s socioeconomic status.) Although the use of electricity and kerosene was almost similar across all wealth quintiles, over 90% of the three lowest wealth quintiles used wood as their primary cooking fuel, while only 24.5% of the wealthiest quintile used wood [5]. 

Most of the cooking in Sri Lankan households is done by women, and keeping company with these women are their young, pre-school children and elderly members of their families. These cultural practices, coupled with the nature of Sri Lanka’s demographics, with sizable populations of very young and also the aged (unusual in a lower-income country), suggest that large segments of its population are exposed to air pollutants inside their homes during biomass stove operation.

### 3.3. Health Risks Associated with Biomass Fuel Stove Use

Traditional biomass fuel stoves produce particulates, carbon monoxide, nitrous oxide, sulfur oxides, formaldehyde and polycyclic organic matter, which includes carcinogens such as benzopyrene [11]. Particulates, especially PM_2.5_, are harmful because they penetrate deep into the lungs. These particulates cause bronchial irritation, inflammation and fibrosis and can lead to wheezing, exacerbation of asthma, respiratory infections and chronic obstructive pulmonary disease (COPD) [11]. Carbon monoxide prevents hemoglobin from delivering oxygen to key organs and the developing fetus [12]. Nitrogen dioxide and sulfur dioxide increase bronchial reactivity and lead to wheezing, asthma and more serious respiratory problems like lung infections and COPD. Formaldehyde irritates the nasopharyngeal region, may lead to asthma and is also a probable carcinogen. Benzopyrene is a carcinogen and causes lung cancer. Biomass smoke also causes oxidative changes to the eye’s lens and leads to cataracts [11]. Recent review of epidemiological studies on indoor air pollution and health has identified six studies in Sri Lanka [13]: These studies were on asthma [14,15], respiratory symptoms [16], low birth weight [17] lung cancer [18] and cataract [19]. Except the study on lung cancer all other studies show a positive association between health outcome and use of biomass for cooking. Another recent study conducted in two different outdoor air pollution settings showed that the use of biomass was a risk factor for wheezing independent of the area of residence (adjusted odds ratio (OR): 1.57; 95% confidence interval (CI) = 1.01–2.46) [20] 

### 3.4. Sri Lanka Health Data Associated with Biomass Fuel Stove Exposure

Sri Lanka’s data and government reports do not attribute adverse health effects to biomass fuel use. However, the morbidity and mortality pattern of Sri Lankan health data are suggestive of adverse health effects including chronic and acute respiratory diseases, cardiovascular diseases, cancers *etc*. of exposure to indoor air pollutants from biomass smoke. 

Sri Lankan public hospital statistics indicate that respiratory disease commands a large portion of the country’s public health expenditures, remaining one of the leading causes of hospitalization among all age groups. In fact, in the 2007 edition of the Sri Lankan Annual Health Statistics Bulletin, the category “diseases of the respiratory system” was the second leading cause of hospitalizations in 2007 when averaged across all 25 districts. Sri Lanka government data show that “cancer of the trachea and lungs,” was the fifth leading type of cancer for 2005 [21]. Although, in-country evidence not available to date, exposure to biomass smoke is known to cause lung cancers [22]. 

Additionally, diseases of the circulatory system, which include all types of heart disease, had relatively high mortality rates for young children less than 1 year old (85.6) and high rates for elderly (598.4 for 60–69 and 1,449.8 for 70+) per 100,000 people in the population. Hypertensive diseases and other circulatory diseases are each in the top 20 causes of hospitalization in Sri Lanka [23]. 

### 3.5. Vulnerable Populations

The poor and less-educated live in rural areas are at the greatest risk for IAP-related health problems. They are the least likely to use cleaner fuels or a ventilated kitchen because the costs are prohibitive. In particular, children, women and the elderly typically are present in the home during preparation of meals, which means they are also more exposed to the airborne particles produced by burning biomass. 

Respiratory diseases and pneumonia have resulted in higher mortality rates in infants and children under the age of 5 years. Respiratory disease caused 9% of all deaths in children between the ages of 1 to 4 years old [23]. Although Sri Lanka’s respiratory infection death rates are lower than South-East Asian regions, they remain high.

Moreover, there continues to be a wide disparity in infant mortality rates from district to district; these disparities are reflective of poverty-related neonatal and maternal illnesses, access to health care across districts and differential under-reporting, especially for northern districts affected by the civil conflict and, more recently, eastern districts affected by the Pacific tsunami [24].

In Sri Lanka, exposure to biomass smoke puts pregnant women and their unborn children at risk. In 2007, infant mortality per 1000 live births was 10 in urban areas, 19 in rural areas, and 29 on estates [1]. Biomass smoke impairs fetal development, and increases the risk of miscarriages, preterm births stillbirth and low birth weight [25,26]. Recent estimates indicate that 17% of Sri Lankan infants are born with a low birth weight [27]. A study conducted in three districts of Sri Lanka reported that the low birth weight is associated with the use of wood compare to clean fuels (*i.e*., gas or electricity) (OR = 3.9, 95% CI = 1.8–8.5) [17].

Sri Lanka includes a large proportion of elderly (9.2%) over the age of 60. Many of the elderly, more than 77%, live with their children [28]. Elderly women, who spend their time in cooking indoors, are being exposed to IAP produced by the cook stoves. These women are at increased risk for developing cataracts, heart disease, respiratory diseases *etc*. [11,29,30] A study reported an association (*p* < 0.05) between biomass exposure and cataract by comparing cataract patients (cases, *n* = 197) with patients admitted for other eye problems (controls, *n* = 190) to the National Eye Hospital, Colombo [19]. 

### 3.6. Costs

IAP is likely to be a significant burden on the public health system in Sri Lanka. As an example, WHO estimates the number of deaths attributable to IAP in Sri Lanka in 2004 was 4,300 compared to number of deaths attributable to diarrhea and outdoor air pollution were 800 and 1000 respectively [31]. Estimated disability adjusted life years due to IAP in the same year was 3 per 1000 *capita* per year. Probably the actual burden is higher than the WHO estimates as this was based on the regional predicted solid fuel use of 67% [32] while the actual use of solid fuel in Sri Lanka exceed 78% [1]. There are significant healthcare costs associated with biomass cook stove use, as well as additional negative factors associated with fuel collection (risk to women walking along, time spent collecting wood, risk of snake bites during wood collection, *etc*.) [32]. Actual costs of the biomass fuel use or IAP have not been estimated in Sri Lanka.

### 3.7. Measuring Air Pollution in Sri Lanka

Despite the significant risks associated with biomass fuel stove use, there are limited IAP data available, and few research studies have measured indoor air quality levels in Sri Lankan households. A study assessing exposure in rural kitchens (*n* = 10) using wood with traditional stoves reported 24 h average PM_2.5_ concentrations exceeding 1200 μg/m^3^ [9]. These PM_2.5_ concentrations are excessive; WHO’s recommended guideline for ambient levels of PM_2.5_ is a 24 h mean of 25 µg/m^3^ [33]. From 2009 to 2010, researchers conducted a children’s longitudinal exposure study (*n* = 612) in urban and semi-urban settings in Sri Lanka. The urban area, in Colombo, reportedly has high levels of ambient air pollution, and the semi-urban area, in the Panadura Medical Officer of Health area, was thought to have much lower levels of ambient air pollution. PM_2.5_ was measured in the primary living area by UCB particle monitors and outside the homes by GENT gravimetric air samplers in a sub-sample of households (*n* = 198). Measurements were made inside the living area. Indoor PM_2.5_ measurements from 132 urban homes had a mean of 84.4 µg/m^3^ and a range of 25.2 µg/m^3^ to 620.6 µg/m^3^. The 66 rural homes had a mean of 94.4 µg/m^3^ and a range of 5.9 µg/m^3^ to 755.0 µg/m^3^. The highest indoor PM_2.5_ measurements were reported from homes that burned biomass for cooking fuel in urban setting (mean of 243.2 µg/m^3^ and a range of 40.7 µg/m^3^ to 620.6 µg/m^3^). Respiratory symptoms of each child were monitored by daily respiratory symptom diaries collected for one year. Children who were exposed to high indoor PM_2.5_ (RR = 2.2 [CI: 1.8–2.6]) and living in an urban setting (exposure to high outdoor PM_2.5_) (RR = 1.3 [CI: 1.1–1.6]) reported wheezing more frequently when adjusted for other potential risk factors by poisson regression [2]. Another study conducted in an urban location of Sri Lanka (a Colombo suburb), reported that the respirable dust levels in 84% of households using firewood and in 54% of households not using firewood exceeded the WHO standards [16]. 

Despite many discussions to expand outdoor air quality monitoring in Sri Lanka, there has been only one station to monitor ambient air quality on a continuous basis since January 1997 to date. Sri Lanka’s Central Environment Authority (CEA) monitors and operates this real-time ambient monitor in the city of Colombo (located at Colombo Fort). An inter-country comparison of PM_10 _based on 24 h measurements on weekly basis for 3 years from year 2002 has reported using the same methodology and instruments. The reported 3 year average value from Colombo Fort (an urban location) was 73.37 μg/m^3^ and in a residential area of Colombo was 58.82 μg/m^3^. The values reported from other regional locations were: Bangladesh urban location: 45.76 μg/m^3^; India-Trombay: 37.34 μg/m^3^; India-Vashi: 82.83 μg/m^3^; Pakistan: 67.45 μg/m^3^, Thailand, urban: 38.67 μg/m^3^, Thailand, suburban: 25.77 μg/m^3^ and Vietnam: 50.29 μg/m^3^ [34]. Ambient air quality data from rural Sri Lankan locations are scare.

### 3.8. Governmental and Nongovernmental Institutions

A number of national agencies are involved in aspects of air pollution regulation and monitoring in Sri Lanka. The development of policies and programming focused on IAP is largely centralized at the national level. Cities and local governments have shown limited interest or have not developed expertise in this area. 

#### 3.8.1. National Agencies

The Ministry of Environment and Natural Resources (ME & NR) has lead responsibility for environmental management in the country. Within ME & NR, the Air Resources Management Center (AirMAC) is established to provide leadership for policy and regulatory issues related to air pollution monitoring and control. 

The CEA was established in 1981 under the National Environmental Act of 1980 and pre-dates the establishment of the ME&NR, created in 2001. ME&NR has the overall responsibility for CEA. CEA has the mandate to monitor ambient air quality and responsibility for dissemination of data from its air quality monitoring program. The permissible ambient air quality standards for selected air pollutants were established under the National Environmental (Ambient Air Quality) Regulations of 1994. The standards were modified in August 2008 in reference to the WHO guidelines of 2005 [33]. National policies to assess and reduce IAP are few. The enactment of the National Authority on Tobacco and Alcohol Act (2006) is an attempt to reduce exposure to second-hand smoke in public places. The Act banned smoking in healthcare institutions, educational institutions, government facilities, universities, indoor offices and other indoor workplaces. However, there are no indoor air quality standards enacted to date. 

Under the Ministry of Power and Energy, the Sustainable Energy Authority was established in 2007 and operates with a mandate to ensure energy security, promote indigenous energy use and promote energy efficiency. Energy-efficient cookstoves are identified as one of their program priorities. 

The Ministry of Health is responsible for the nation’s public health and carries a mandate to support the health status of citizens through health promotion as well as preventive, curative and rehabilitative services. The health system in Sri Lanka has both public and private healthcare services. The public sector provides preventive health services that cover the country via the health administrative divisions. The Directorate of Environmental and Occupational Health (DEOH) acts on issues of environmental health at the national level. Public education and awareness-raising programming related to IAP are the responsibility of the DEOH. 

The National Building Research Organization (NBRO) operates under the Ministry of Disaster Management and Human Rights. The NBRO Environmental Division maintains a well-equipped laboratory for ambient air quality testing. 

#### 3.8.2. University Actors in Sri Lanka

Most of the research projects are part of fulfilling an academic degree, and findings are typically confined to conference presentations/abstracts. Few studies have been published in local journals or are available electronically. Several of Sri Lanka’s leading universities have active research initiatives related to IAP. As examples, University of Kelaniya has collaborated with the University of Alabama at Birmingham, USA; and University of Moratuwa has collaborated with the Polytechnic University of Hong Kong to initiate IAP studies.

#### 3.8.3. Nongovernmental Organization (NGO) Actors

Several non-profit and community organizations are active in Sri Lanka on issues of IAP and improved cookstove development.

The Integrated Development Association (IDEA), based in Kandy, Sri Lanka, is an NGO focused on promoting sustainable development and natural resource management. One of IDEA’s programs was the development, testing and promotion of the Anagi indoor cookstove, which is a two-pot, one-piece clay cookstove. The word “Anagi” means precious or excellence in the Sinhala language. Anagi was first introduced in 1986 by the Ceylon Electricity Board in collaboration with the Intermediate Technology Development Group (ITDG, now Practical Action [PA]) under the Urban Stoves Program. Its success prompted the stove to be selected for commercialization in the rural areas with the participation of IDEA and ITDG. 

In the past, IDEA also implemented a Kitchen Improvement Program in Sri Lanka, with the support of Asian Region Cookstove Program and the United Nations Development Programme Global Environment Facility. IDEA has measured the PM_2.5_ and CO levels in the kitchens where they have completed interventions. (PM_2.5_ levels were measured using the UCB particle monitors, and CO was measured using HOBO CO monitors.) IDEA has also managed small and medium enterprise projects to improve brick kilns, in an effort to make the kilns more efficient and reduce firewood consumption. 

PA works to reduce IAP by helping villages to share ideas about cooking in a safe manner. The NGO has installed smoke hoods and fuel-efficient or bottled gas stoves; facilitated improvement in building design, such as large eave spaces under the roof and small windows to improve ventilation; and improved stove design to reduce firewood demand. PA’s projects are often conducted through partner organizations, on a very small scale. PA has initiated a project to assess the health effects and IAP caused by biomass fuel in the rural estate sector of the country, in partnership with the University of Moratuwa. 

#### 3.8.4. International Donor Actors

Several international donors are active in supporting Sri Lanka with financial and technical assistance in the areas of energy, environment and public health. Major donors include ADB, the World Bank, the United Nations organizations and the US Agency for International Development (USAID).

WHO organized a regional workshop on indoor air pollution and household energy monitoring in 2006 in Sri Lanka. Further WHO had allocated limited funds to Ministry of Health for conducting awareness programs on the IAP issue to health professionals. 

ADB has a broad program working on issues of energy, environment and health, among other sector priorities. Biomass stoves or IAP issues have not been a specific focus of their projects in Sri Lanka, although the issue is referenced as a regional concern. 

The World Bank has a broad program working on issues of energy, environment and health, among other sector priorities. Biomass stoves or IAP issues have not been a specific focus of their recent projects. 

USAID/Sri Lanka has worked in the past with private-sector partnerships to leverage resources to address environmental pollution. USAID, through its Asia regional environmental project known as “ECO-Asia,” recently released a 2010 report on black carbon in the Asia region. 

Partnership for Clean Indoor Air (PCIA) is a broad network of 365 organizations worldwide working on issues of improved stoves and IAP. PCIA garners financial support from several organizations, including the US Environmental Protection Agency, to fund the network’s Secretariat. 

## 4. Conclusions

Nearly 80% of Sri Lanka’s population still relies on biomass fuel. Improved cookstoves have been disseminated in Sri Lanka to a limited extent, but their effectiveness of reducing emissions has not been determined. IAP from biomass is likely to be a major public health problem in Sri Lanka based on the limited in-country evidence and the findings from other countries. However, further studies are necessary to explore indoor air quality levels, exposure to biomass fuel and epidemiological studies on biomass exposure and health effects. While the shifting from biomass to clean fuels is challenging in reality, it is imperative to take actions to reduce the IAP. With a new focus on IAP, Sri Lanka is well equipped to tap its strong public health system and government infrastructure to address IAP from biomass fuel use. A combination of technology, education and outreach that is culturally appropriate, affordable and convenient will be critical factors for successful programming. New global initiatives, such as the Global Alliance for Clean Cookstoves (GACC), provide valuable advocacy, and may potentially provide financial resources, to advance understanding of environmental risks and pursue new programming to reduce the risks from IAP.
